# Correction Method for Optical Scaling of Fundoscopy Images: Development, Validation, and First Implementation

**DOI:** 10.1167/iovs.65.1.43

**Published:** 2024-01-25

**Authors:** Lennart J. Pors, Corné Haasjes, Luc van Vught, Noor P. Hoes, Gregorius P. M. Luyten, Gwyneth A. van Rijn, T. H. Khanh Vu, Coen R. N. Rasch, Nanda Horeweg, Jan-Willem M. Beenakker

**Affiliations:** 1 Department of Radiation Oncology, Leiden University Medical Center, Leiden, the Netherlands; 2 Department of Ophthalmology, Leiden University Medical Center, Leiden, the Netherlands; 3 Department of Radiology, Leiden University Medical Center, Leiden, the Netherlands; 4 Department of Ophthalmology, Amsterdam University Medical Center, Amsterdam, the Netherlands; 5 Department of Ophthalmology, Northwest Clinics, Alkmaar, the Netherlands

**Keywords:** fundus photography, refraction, optical scaling, myopia, uveal melanoma

## Abstract

**Purpose:**

Although fundus photography is extensively used in ophthalmology, refraction prevents accurate distance measurement on fundus images, as the resulting scaling differs between subjects due to varying ocular anatomy. We propose a PARaxial Optical fundus Scaling (PAROS) method to correct for this variation using commonly available clinical data.

**Methods:**

The complete optics of the eye and fundus camera were modeled using ray transfer matrix formalism to obtain fundus image magnification. The subject's ocular geometry was personalized using biometry, spherical equivalent of refraction (RSE), keratometry, and/or corneal topography data. The PAROS method was validated using 41 different eye phantoms and subsequently evaluated in 44 healthy phakic subjects (of whom 11 had phakic intraocular lenses [pIOLs]), 29 pseudophakic subjects, and 21 patients with uveal melanoma.

**Results:**

Validation of the PAROS method showed small differences between model and actual image magnification (maximum 3.3%). Relative to the average eye, large differences in fundus magnification were observed, ranging from 0.79 to 1.48. Magnification was strongly inversely related to RSE (*R*^2^ = 0.67). In phakic subjects, magnification was directly proportional to axial length (*R*^2^ = 0.34). The inverse relation was seen in pIOL (*R*^2^ = 0.79) and pseudophakic (*R*^2^ = 0.12) subjects. RSE was a strong contributor to magnification differences (1%–83%). As this effect is not considered in the commonly used Bennett–Littmann method, statistically significant differences up to 40% (mean absolute 9%) were observed compared to the PAROS method (*P* < 0.001).

**Conclusions:**

The significant differences in fundus image scaling observed among subjects can be accurately accounted for with the PAROS method, enabling more accurate quantitative assessment of fundus photography.

Fundoscopic imaging is one of the most frequently used methods to assess the interior structures of the eye. In addition to visualizing retinal pathology such as diabetic retinopathy,^1^ the resulting images can also be used to measure the distance between retinal structures. For example, in ocular oncology the distances between the optic disk, macula, and tumor are used in radiation treatment planning^2^^,^^3^ (Fig. 1A). Additionally, lesion size is measured to determine disease progression in patchy chorioretinal atrophy^4^ (Fig. 1B), and optic disk size is used in the assessment and diagnosis of glaucoma patients.^5^^,^^6^ However, as these images are formed by light rays that pass through the lens and cornea, changes in their optical properties will result in differences in magnification of fundus images,^7^ as illustrated in Figure 1C. As a result, fundus photographs cannot be used for accurate distance measurements, unless they are corrected for these optical effects.

**Figure 1.**
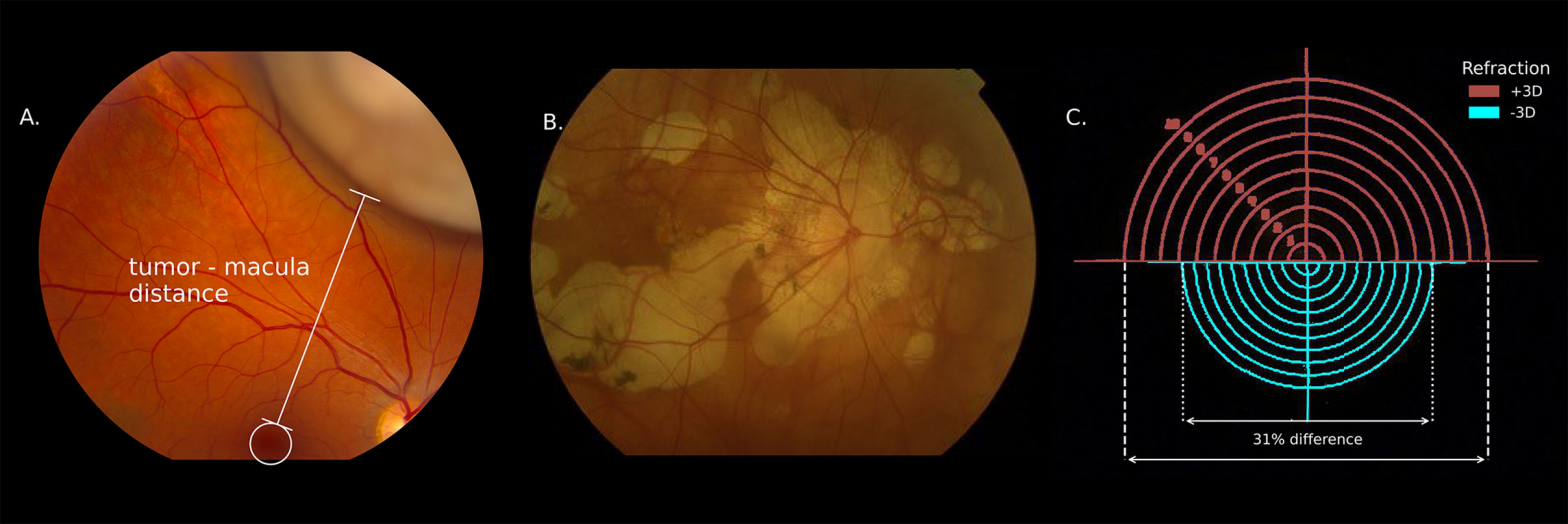
Fundus photography is a widely used imaging technique to, for example, visualize an intraocular tumor (**A**) or patchy chorioretinal atrophy (**B**). The scale of these photographs differs between subjects, as is illustrated by fundus photographs of the same object in two phantom eyes with a different refraction (**C**).

In the 1980s, Littmann and Bennett proposed a method to correct for this scaling difference in fundus photographs acquired with a specific Zeiss fundus camera^8^^–^^10^:

$$\begin{array}{c} d_{true}=c_{camera}*\left(0.01306*\left(AL-1.82\right)\right)*d_{camera} \end{array}$$

where *d_true_* is the true retinal size of an object, *c_camera_* is a camera-specific constant, *AL* is the axial length of the eye, and *d_camera_* is the image size on the camera. Thus, in the Bennett–Littmann method, the image magnification scales linearly with the axial length.

Although the Bennett–Littmann method has been widely used^11^^–^^13^ and has significantly improved the accuracy of fundus-based measurements, it has some limitations. First, the method is based on the telecentric camera design employed by Zeiss in the 1980s.^8^ Telecentricity implies that changing the camera focus, which is required to correct for the patient's refraction, has no effect on the image size.^14^ However, current fundoscopy cameras are often not telecentric,^15^ invalidating the assumption that scaling factor *d_camera_* is independent of the patient's refraction. Second, the method is inaccurate for ametropic eyes, as the magnification caused by the converging or diverging light rays between the eye and camera in these patients is ignored. Third, although the method corrects for differences in axial length, which have the strongest impact on magnification, differences in other anatomical elements such as corneal curvature are neglected.^16^ Finally, the Bennett–Littmann method is not applicable to eyes with an intraocular lens (IOL), as ocular optics are changed by lens implantation.^17^ The impact of these limitations is currently not known, as the Bennett–Littmann method has not been thoroughly validated.

In this study, we aimed to resolve these limitations of the Bennett–Littmann method by developing a patient-specific optical model of the eye and camera: the PARaxial Optical fundus Scaling method (PAROS). We extensively validated this model with different optical eye phantoms and sequential ray tracing. Subsequently, the differences in image scaling between the PAROS method and the Bennett–Littmann method in eyes with and without a phakic or pseudophakic IOL and the impact of potential limitations of the Bennett–Littmann method were assessed. Finally, we explored the possible clinical impact of the PAROS method on optic disk measurement and uveal melanoma treatment planning. We hypothesized that accounting for the abovementioned limitations in a full optical model of the eye and camera will result in significantly more accurate measurements on fundus photographs.

## Methods

A mathematical model was built to describe the paraxial optics of the eye and fundus camera. Subsequently, the parameters of the camera model were calibrated using an adjustable eye phantom and thereafter the complete method was validated. Finally, the method was applied in four case series and compared to the Bennett–Littmann method.

### Optical Model

The propagation of light rays from the retina through the eye and camera was calculated using the ray transfer matrix formalism^18^ with SymPy 1.10.1, a Python library for symbolic mathematics.^19^ In the ray transfer matrix formalism, the refraction of light rays (for example, at the lens–vitreous interface) is described by a 2 × 2 matrix whose elements depend on the radius of curvature of the interface and on the refractive indices of both media. This method has been used in ophthalmological research before.^20^ The complete camera and eye system can be described by multiplication of the matrices of the individual optical elements. A full mathematical description of the model, the main elements of which are described below, can be found in the Appendix, and the corresponding code is available at https://github.com/MREYE-LUMC/PAROS (Fig. 2).

**Figure 2.**
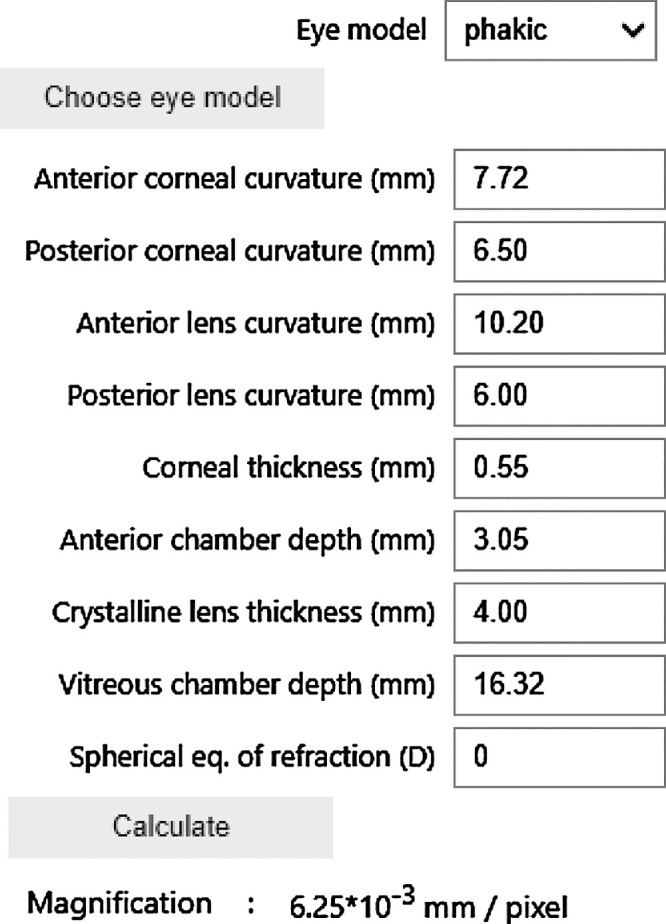
Screenshot of the user interface of PAROS, which can be accessed via https://github.com/MREYE-LUMC/PAROS, where the full PAROS code can also be obtained.

The eye model was based on the Escudero-Sanz–Navarro widefield model,^21^ in which the corneal curvatures, corneal thickness, anterior chamber depth, lens thickness, and axial length can be personalized. A wavelength of 543 nm and corresponding refractive indexes were used.^21^ For pseudophakic subjects, the clinical IOL model of van Vught et al.^22^ was used with a refractive index of 1.47 and a thickness of 1 mm. The posterior curvature of the lens or IOL was chosen such that the refraction of the eye model matched the spherical equivalent objective refraction (RSE) of the subject. For phakic intraocular lenses (pIOLs), the lens curvatures were based on the International Organization for Standardization standard ISO 11979-2:2014,^23^ with a thickness of 0.2 mm and *n* = 1.47.

The optical setup of the fundus camera was approximated by two thin lenses: a condenser lens and a focusing lens. The radius of curvature of the focusing lens was chosen such that it produced a sharp image of the retina on the image plane, which was set at the focal point of the condenser lens. As this is a simplification of the actual camera, which contains multiple thick lenses that can move to correct the focus of the camera, a first-order calibration term, which depended on the radius of the focusing lens, was added. This resulted in the following ray transfer matrix of the camera:

$$\begin{array}{c} \left[\begin{array}{cc} -\frac{F_{cond}\left(r_{foc}+a_{1}\right)}{r_{\mathrm{foc}}^{2}} & \frac{F_{cond}\left(r_{foc}+a_{1}\right)}{r_{foc}}\\ -\frac{F_{cond}+r_{foc}}{F_{cond}\left(r_{foc}+a_{1}\right)} & \frac{r_{foc}}{r_{foc}+a_{1}} \end{array}\right] \end{array}$$

where *F_cond_* is the effective focal length of the camera, *r_foc_* is the radius of curvature of the focusing lens, and *a*_1_ is the first-order calibration term.

Implementation of the eye and camera model was validated using sequential ray tracing in OpticStudio 2023 R1.00 (Ansys, Canonsburg, PA, USA) with ZOSPy 1.1.0^24^ for 50 randomly generated eye models.

### Camera Calibration and Eye Phantom Validation

To calibrate the camera and subsequently validate the complete methodology, an eye phantom was developed (Fig. 3). The phantom consisted of lens tubes (SM1L20 and SM1L15; Thorlabs, Newton, NJ, USA), in which 1-inch lenses could be accurately positioned. At the back end of the lens tube, an optical calibration phantom was positioned that consisted of 10 1-mm-spaced, concentric circles (R1DS2N; Thorlabs), hereafter referred to as the calibration object. To limit interfering environmental light, a 2-mm pinhole was mounted in front of the first lens. The eye phantom could be mounted on the chin rest of the fundus camera with a distance of 5 cm between the first lens and the condenser lens.

**Figure 3.**
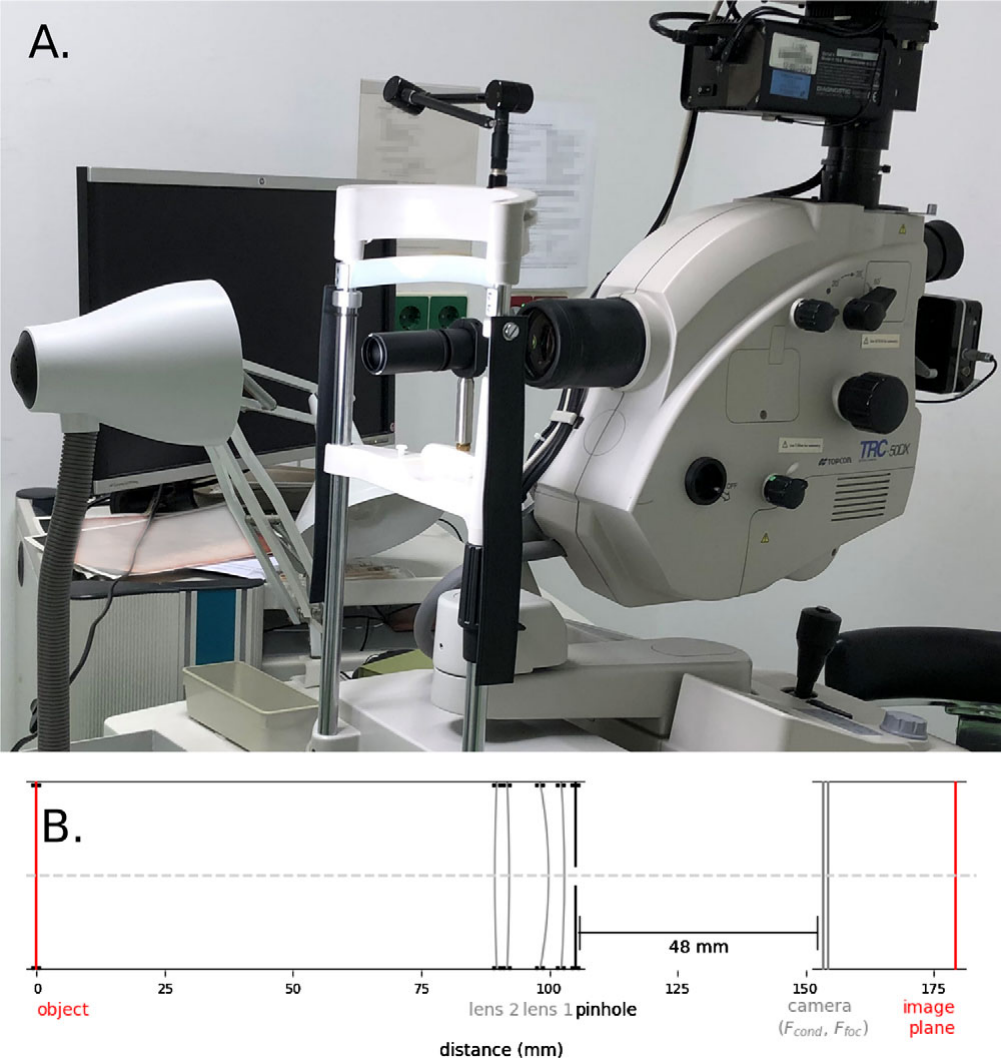
The phantom eye setup. (**A**) Overview of the setup consisting of a lens tube in which a calibration object is illuminated from the back. The phantom eye is attached to the chin rest of the fundus camera (Topcon TRC-50DX) for reliable positioning. (**B**) Schematic drawing of the setup for phantom eye 20, with a 5-D biconvex lens representing the eye lens and an 8-D convex–concave lens representing the cornea.

**Figure 4.**
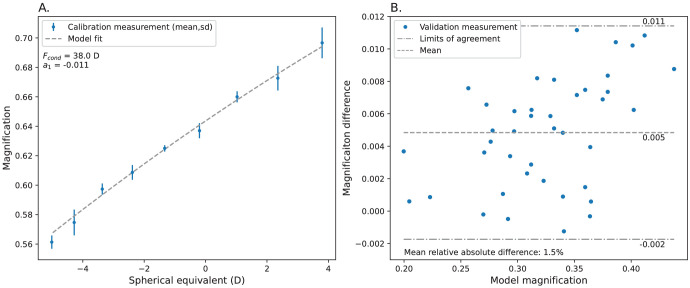
Calibration and validation measurements of the eye and camera model. (**A**) The optical parameters of the camera model were obtained by measuring the magnification for nine different eye models. This procedure was performed three times to show the high reproducibility of the method. (**B**) The complete method was validated for 41 phantom eyes with a wide range of optical characteristics that showed small (on average, 1.5%) differences with the actual size of the calibration target.

To measure the effective focal length of the fundus camera and the first-order correction term of the model, an eye phantom was constructed with a 20-D biconvex lens (LB1471; Thorlabs) and a 5-D biconvex lens (LB1945; Thorlabs), spaced 3 mm apart. Nine different positions of the calibration object were used, ranging in distance from 29.5 mm to 45.5 mm from the second lens, resulting in refractive errors ranging from −5.0 to +3.8 D. For each setup, the diameter between the outer circles of the calibration object was measured and compared to the physical diameter (10 mm) to obtain the magnification. The same setup was also modeled using ray transfer matrices, and the optimal *F_cond_* and *a*_1_ were determined using a Nelder–Mead minimizer in SciPy 1.9.1.^25^

Both the calibration and validation were performed on a TRC-50DX fundoscopy camera (Topcon, Tokyo, Japan). The reproducibility of the complete calibration procedure was determined by having it performed three times by two different persons (LJP, CHA). For subsequent analyses of this camera, the average of the three camera constant measurements was used.

Subsequently, the complete method was validated with an additional set of 41 phantoms, which consisted of a convex–concave lens resembling the cornea and a biconvex lens resembling the crystalline lens of the eye. In total, three different convex–concave lenses (LE1234, LE1156, LE1104; Thorlabs) and three different biconvex lenses (LB1056, LB1779, LB1945; Thorlabs) were used, and wide ranges of locations for both the lenses and the calibration object were applied. In total, these 41 phantoms covered refractive errors ranging from −8.3 to +4.2 D. A complete list of the phantoms used and corresponding measurements can be found in the Supplementary Materials.

### Clinical Evaluation

The effect of different ocular geometries on the magnification of fundus images was evaluated for phakic, pseudophakic, and pIOL eyes without known ocular pathologies (except the earlier performed, uneventful lens implantations for the [p]IOL groups) and in eyes with uveal melanoma, the most common primary intraocular tumor in adults.^26^ For the healthy eyes, these data had been acquired as part of earlier scientific studies.^24^^,^^27^^–^^30^ For the uveal melanoma eyes, 48 consecutive patients treated with proton beam therapy who had given written informed consent for the retrospective use of their data were included. From these patients, 19 were excluded due to missing refraction data, six due to incomplete biometry data, and two due to astigmatism of >3 D. This resulted in 33 phakic, 29 pseudophakic, 11 pIOL, and 21 uveal melanoma subjects. An overview of the demographics of all patient groups can be found in the Table.

**Table.** Descriptive Statistics for the four case series

	Subjects			
	Healthy (*n* = 33)	Pseudophakic (*n* = 29)	pIOL (*n* = 11)	Uveal Melanoma (*n* = 21)
Sex (male), *n* (%)	22 (33)	15 (52)	4 (36)	10 (48)
Eye (OD), *n* (%)	2 (6)	15 (52)	9 (82)	14 (67)
Age (y), median (range)	26.5 (17–74)	68.8 (48–80)	51.9 (36–65)	60.7 (25–73)
Pseudophakic, *n* (%)	0 (0)	29 (100)	0 (0)	0 (0)
RSE (D), median (range)	−1.25 (−7.1 to 1.6)	−0.4 (−3.6 to 0.8)	−0.125 (−2.5 to 1.4)	0.3 (−5.8 to 5.1)
Axial length (mm), median (range)	23.64 (22.4–26.8)	24.0 (20.5–28.4)	23.1 (20.5–30.4)	23.6 (21.7–26.7)
Anterior corneal curvature (mm), median (range)	7.8 (7.2–8.3)	7.7 (7.1–8.2)	7.8 (7.6–8.3)	7.8 (7.4–8.6)
Corneal thickness (mm), median (range)	0.55 (0.47–0.60)	0.55 (0.47–0.62)	0.56 (0.51–0.61)	0.56 (0.50–0.64)
Anterior chamber depth (mm), median (range)	3.2 (1.9–3.8)	4.3 (3.9–4.7)	2.1 (1.3–2.5)	2.6 (2.1–3.5)
Lens thickness (mm), median (range)	3.7 (3.2–5.3)	—	4.9 (3.9–5.3)	4.6 (3.5–5.3)

For each eye, the fundus image magnification was calculated using the PAROS method, and the magnification according to the Bennett–Littmann method was calculated, as well. For the phakic and uveal melanoma subjects, the complete ocular geometry was based on data from the Lenstar LS900 biometer (Haag-Streit AG, Köniz, Switzerland). The radius of the posterior corneal surface was defined as 0.81 times the measured radius of its anterior surface.^31^ For the pseudophakic and pIOL groups, only the axial length and posterior lens surface location were obtained from the Lenstar. All other variables, including the posterior radius of the cornea for the pseudophakic eyes, were obtained with a Pentacam anterior segment tomographer (software version 1.20r41; OCULUS, Optikgerate GmbH, Wetzlar, Germany). The RSE was obtained with an autorefractor. In two pIOL subjects, no autorefractor measurement was performed and the subjective RSE was <0.5, so RSE was assumed to be 0. Finally, in order to calculate the magnification according to the Bennett–Littman method, the camera-specific constant *c_camera_* was calibrated using the Navarro eye model.

We explored the clinical relevance of the proposed scaling correction of fundus photographs for two clinical applications: tumor-macula distance in radiotherapy planning and lesion size estimation in patchy chorioretinal atrophy in pathologic myopia.^4^ Currently, the tumor location and extent in ocular proton beam therapy are primarily modeled using tantalum clips and surgical measurements.^32^ One of the steps toward clipless treatment would be to use fundus photographs to determine the tumor location relative to the macula. We assessed the impact of the varying scale of fundus images for a patient with an apparent 12.5-mm tumor–macula distance (Fig. 1A) by calculating the probability distribution of true tumor–macula distance using the data of all subject groups combined (except for the pIOL eyes as these are not abundant in the normal population). As a threshold for a clinically relevant error, the currently used 1.0-mm margin for setup error was used.^33^ For the second clinical application, we assessed the impact of myopization on the apparent size of retinal pathologies. In the study by Ruiz-Moreno et al.,^4^ which investigated patchy chorioretinal atrophy in highly myopic patients, a mean annual increase in lesion size of 71% was found. We assessed the potential confounding effect of the further myopization of these subjects on the observed increase in lesion size, using the annual change in refraction of a cohort of 154 highly myopic subjects as reported by Verkicharla et al.^34^

## Results

The results of the camera calibration, eye phantom validation, and sequential ray-tracing validation are presented first. Thereafter, the magnification is calculated for the pooled patient group, followed by a breakdown of the impact of different parts of the model on total magnification. Finally, the clinical impact of correcting fundus measurements using the PAROS method is discussed.

### Model Validation and Camera Calibration

The magnifications obtained with the proposed paraxial ray transfer methodology were validated to full optical ray-tracing simulations in 50 eyes with a randomly varying geometry. The absolute relative difference between both models was below 0.1% for all eyes, which had RSEs ranging from −15 D to +10 D. The calibration was performed in total three times by two observers on nine eye phantom calibration setups. The resulting mean power of the condenser lens was 38.0 D (SD = 0.2 D); for the first-order calibration term, it was −0.011 (SD = 0.002). The charge-coupled device (CCD) was thus located at 26 mm behind the condenser lens, at its focal point. This calibrated eye–camera model yielded a magnification of 1.60 for the Navarro eye. In the validation set of 41 eye phantoms, the proposed method resulted on average in a 1.5% overestimation of the magnification, with all absolute differences below 3.3% (Fig. 4).

### Model Application

The groups with phakic, pseudophakic, and uveal melanoma affected eyes were pooled to calculate magnification, resulting in 94 eyes of which 29 were pseudophakic and 65 phakic. The variations in age (17–80 years), RSE (−7.1 to 5.1 D), axial length (20.5–30.4 mm), corneal topography, and anterior chamber depth were substantial. There were some differences between the groups. The mean ages in the uveal melanoma and pIOL groups were higher than in the phakic group, explaining the thicker lenses in these patients. As expected, the anterior chamber was deeper than average in the pseudophakic subjects and less deep in the pIOL subjects.

The magnification relative to the average eye ranged between 0.79 and 1.48. Relative magnification was inversely proportional to spherical equivalent (*R*^2^ = 0.67) (Fig. 5A) and was not related to axial length in the complete group (*R*^2^ = 0.002) (Fig. 5B). This relation to axial length was directly proportional in phakic (*R*^2^ = 0.34), but inversely proportional in pseudophakic (*R*^2^ = 0.12) and pIOL (*R*^2^ = 0.79) subjects. Relative magnification as corrected by the PAROS method was statistically significantly different to no correction (*P* < 0.001) and to the Bennett approximation (*P* < 0.001; mean absolute difference, 9%; range, −38 to 40%) in paired samples *t*-tests.

**Figure 5.**
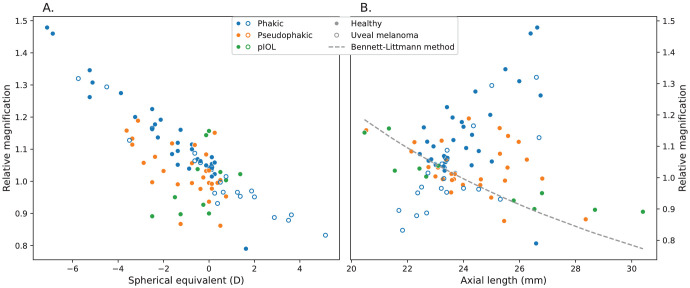
Relative magnification, as calculated by the PAROS method, compared to magnification of the average eye as a function of spherical equivalent (**A**) and axial length (**B**) for different subject groups. Relative magnification ranged from 0.79 to 1.48 and was negatively correlated to spherical equivalent, whereas a less strong positive relation with axial length was observed.

In the model, two distinct contributors to these magnification differences can be discerned: ocular anatomy and camera setup. On average, approximately 47% (range, 1%–99%) of the magnification could be attributed to the ocular anatomy (Fig. 6A). The relation between the magnification caused by the ocular anatomy and axial length is inversely proportional, similar to the method by Bennett et al.^10^ The inclusion of the optical effect of all ocular surfaces as opposed to only axial length led to significant differences between the ocular anatomy–induced magnification in the PAROS method and the total magnification according to the Bennett method (*P* < 0.001, paired samples *t*-test). The other contribution to the magnification differences was the impact of the camera setup, which is influenced by the patient's RSE. This impact was on average 45% of the observed relative magnification (range, 1%–83%) (Fig. 6B) and was inversely proportional to spherical equivalent (and thus directly proportional to axial length).

**Figure 6.**
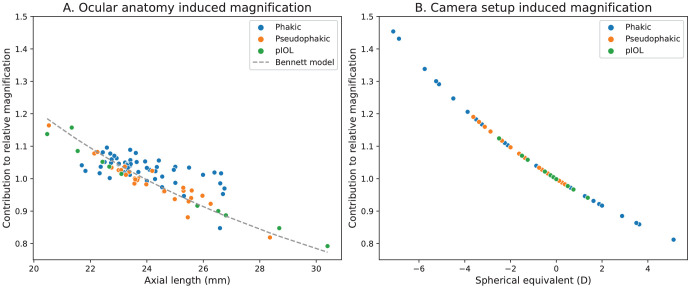
(**A**) The contribution of differences in ocular anatomy to the magnification is relatively accurately described by the Bennett model. (**B**) The camera setup also has a large impact on the magnification. This impact, which is dependent on RSE, is not incorporated in the Bennett model.

### Clinical Impact of Incorrect Fundus Photography Scaling

The clinical relevance of the PAROS method is explored for both tumor–macula distance and optic disk diameter measurements in Figure 7. In Figure 7A, we show the probability of certain corrected tumor–macula distances for the patient of Figure 1A who had an uncorrected distance of 12.5 mm. Given the currently used margins for setup error in ocular proton beam therapy treatment planning (1 mm), there is a 50% probability that the actual distance would fall outside these safety margins. Figure 7B shows the distribution of annual change in fundus magnification due to the further myopization of these patients for the highly myopic cohort from the study of Verkicharla et al.^34^ Due to the change in the eye's optical characteristics, a mean increase of 2.5% (SD = 5.2) in image magnification is observed, which corresponds to approximately 3.5% (SD = 7.3%) of the observed increase in patchy chorioretinal atrophy as described by Ruiz-Moreno et al.^4^

**Figure 7.**
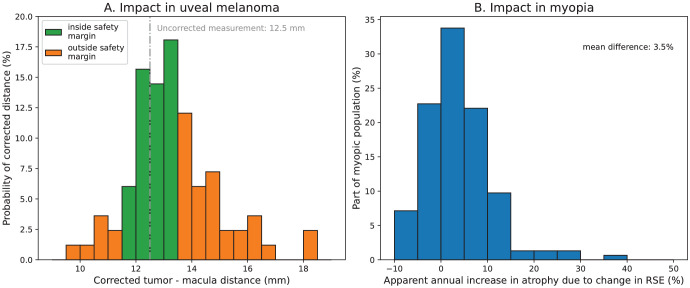
Examples of the potential clinical importance of correcting the varying scaling of fundus images. (**A**) If the 12.5-mm uncorrected tumor–macula distance of Figure 1A is used to localize the tumor, the tumor boundary would be outside the 1-mm safety margin for 50% of the subjects in the study cohort. (**B**) Probability of apparent patchy chorioretinal atrophy lesion size change attributable to RSE change after 1 year, based on the highly myopic cohort of Verkicharla et al.^34^ The probability that >10% of the mean lesion size change is attributable to the RSE change is 15%.

## Discussion

In this study, we showed that the scaling of fundus images can differ up to 50% between patients. These scaling differences can be accurately modeled for paraxial applications using the PAROS method, which is based on the ray transfer matrix formalism. We also showed that this method can be used to correct fundus distances measured on fundoscopy pictures and showed the potential impact in the context of uveal melanoma treatment planning and optic disk categorization.

As PAROS uses a generic camera model, it can easily be adapted to other classic fundus cameras. The eye phantoms used for the calibration are constructed with inexpensive off-the-shelve optical components, which enables a straightforward calibration. For contact-based fundus cameras, such as the Panoret,^35^ one additional modification must be made to the model, as there is liquid instead of air between the cornea and lens, which strongly impacts the refraction at the anterior cornea surface.^36^ However, as the code for PAROS is publicly available at Github (https://github.com/MREYE-LUMC/PAROS), together with a table of known camera calibration constants, such a modification is easily made.

The PAROS method has been validated in two distinct ways. First, the mathematical description, together with the used paraxial approximation and implementation in Python, was validated by comparing the magnification of the method to full sequential ray tracing in OpticStudio for 50 eyes. The negligible (below 0.1%) differences show the mathematical correctness of the developed model. Second, the complete methodology was validated in a set of 41 phantom eyes. The small (below 3.3%) differences between measured and predicted magnification are likely attributable to the difficulty in positioning the lenses inside the phantom eye with submillimeter precision. Although the method can thus be accurately used to assess fundus image scaling, the used ray transfer formalism is only valid for the central retina. For more peripheral measurements, larger differences can be expected, which could be corrected using full sequential ray tracing.^37^

The Bennett–Littmann method uses only the axial length of the eye to calculate magnification.^8^^–^^10^ Due to technological advances in the past three decades, the paraxial optical characteristics of all optical elements of the eye can be included in the proposed mathematical description. As a result, the incorporation of this additional anatomical information leads to a significant improvement of the estimation of the magnification induced by the eye compared to the Bennett–Littmann method. However, the combined modeling of the eye and camera revealed that the patient's RSE is a more relevant source of variation in magnification between subjects, as the camera setup is another main contributor to magnification and is strongly affected by RSE, showing that the camera setup used is not telecentric. Although the effect of ametropia on fundus image scaling was described earlier by Lotmar,^38^ it did not result in an update of the Bennett–Littmann method. The study by Knaapi et al.^11^ constitutes the only in vivo verification of the Bennett–Littmann method. However, when this study is retrospectively evaluated, a negative correlation between prediction error and RSE can be observed, as is expected the light of the PAROS method. The observation that this significant factor contributing to the variation in fundus image scaling has been missed for such a long time emphasizes the importance of robust end-to-end validation.

The PAROS method can be applied in both phakic and pseudophakic eyes, as well as in eyes with a pIOL implant, which is a significant extension compared to the earlier method, which was developed only for phakic eyes. The different associations found for phakic and pseudophakic subjects are caused by the IOL, whose power is selected to correct for the subject's preoperative RSE, breaking the natural dependence of RSE on the axial length of the eye. It was implemented only for eyes with astigmatism <3 D; this method can be easily extended for eyes with larger astigmatism, but in these cases the corneal refraction is dependent on the angle between the cornea and the light rays. The proposed model relies on clinical measurements of the eye, which always have some uncertainties.^30^ However, these uncertainties have limited impact on the calculated magnification, as is shown by increasing the axial length or corneal curvature by 0.1 mm or the RSE by 0.5 D, which resulted in respective 0.5%, 0.6%, and 3.3% changes in magnification for the Escudero-Sanz–Navarro eye model. As the exact optical properties of the lens, including curvature and spatially varying index of refraction, are not easily determined clinically, we fitted the curvature of the posterior lens surface so that the RSE of the eye model would match the clinically measured RSE. Although this will introduce a small error in the anatomical description of the eye, a recent study showed that variations in the optical properties of the lens have a negligible effect on the image scaling.^39^ The most important variable factor in the camera model that cannot be measured is the distance between the eye and the camera. Extending the eye–camera distance by 5 mm led to an absolute magnification change of 1% on average (range, −6% to 2%) in the patient group, which indicates that this uncertainty is fairly small.

In its current implementation, PAROS uses a relatively simple camera model consisting of a condenser lens to calculate the image size on the image plane. This model is not fully representative of optical coherence tomography (OCT) and scanning laser ophthalmoscopy (SLO), as these imaging modalities do not focus light rays onto an imaging plane but instead rely on interference of reflected light rays to visualize anatomy.^36^^,^^40^ As a result, a different dependence on the subject's refraction, the most prominent factor in the magnification of classical fundus cameras, is expected. The PAROS method could be extended to these types of imaging modalities, but doing so would require further validation to ensure accurate mathematical description of the camera. Interestingly, various OCT studies report a similar variation (up to 25%^41^^,^^42^) of image scaling as found in this study (Fig. 6A), suggesting a similar clinically relevant impact of correcting for magnification differences.

The clinical relevance of correcting the scaling of fundus images depends on the measurement performed and the acceptable uncertainties. Because clinicians are aware of the scaling difference on fundoscopy images, these measurements are currently not used independently but are compared with other clinical data, or distances are expressed in amount of optic disk diameters.^31^^,^^43^^,^^44^ This can be ameliorated through dependable correction of fundus scaling. We showed two applications in which correction of fundus photographs can have a significant impact. In the presented uveal melanoma case, there was a 50% probability that the use of uncorrected fundus images would result in clinically significant position errors in radiation therapy planning when no other information would be included to determine the tumor location. In the context of ocular oncology, it is important to note that fundus measurements are always complemented by other modalities such as ultrasound and magnetic resonance imaging.^3^^,^^45^^,^^46^ For highly myopic patients, we showed that in 15% of patients RSE change led to an apparent patchy chorioretinal atrophy lesion size change corresponding to 10% of the mean lesion size change after 1 year, according to Ruiz-Moreno et al.^4^ Although for both applications the actual distribution of magnification differences will depend on the actual clinical population, the overall underestimation of true distances will likely remain due to the increasing prevalence of myopia.^47^

To conclude, the PAROS method is able to correct for the scaling of fundus images and has been validated extensively. The method takes both complete ocular biometry and the camera setup into account, the latter of which is not considered by previous correction methods. In the described case series, magnification differences up to 50% were seen, showing the importance of correction for this scaling issue when fundus distance measurements are taken, such as in ocular radiation therapy treatment planning.

## Supplementary Material

Supplement 1

## Appendix Appendix: Mathematical Description

### Mathematical Description of the Eye and Camera Model

The paraxial light path through the eye and camera is described using ray transfer matrices (see, for example, Chapter 18 of Pedrotti and Pedrotti^18^), with the retina as object and the CCD of the camera as the image plane. As this model relies on the paraxial approximation, all refracting surfaces can be described by their radius of curvature, thus neglecting any higher order components including its conic constant. In the paraxial approximation, the refraction of a light ray at spherical surface, with radius *r*, can be described by the matrix **M**_*sphere*_:

(A.1) $$\begin{array}{ccc} & & \;\;\;\;\;\;\;\;\;\;\;\;\;\;\;\;\;\;M_{\mathrm{sphere}}\left(r,n_{\mathrm{in}},n_{\mathrm{out}}\right)\\ & & \;\;\;\;\;\;\;\;\;\;\;=\left[\begin{array}{cc} 1 & 0\\ \left(n_{\mathrm{in}}-n_{\mathrm{out}}\right)/\left(n_{\mathrm{out}}*r\right) & \left(n_{\mathrm{in}}/n_{\mathrm{out}}\right) \end{array}\right], \end{array}$$

where *n*_in_ and *n*_out_ are the respective refractive indexes of the input and output media. The propagation of a light ray through an uniform medium of thickness *d* can be described by **M**_*unif*_:

(A.2) $$M_{\mathrm{unif}}\left(d\right)=\left[\begin{array}{cc} 1 & d\\ 0 & 1 \end{array}\right].$$

#### Eye Model

The path of light rays through the eye can be described by multiplying the ray transfer matrices of the individual optical elements of the eye: **M**_cor_ for the cornea, **M**_lens_ for the lens, **M**_ACD_ for the anterior chamber, and **M**_vitr_ for the vitreous:

(A.3) $$\begin{array}{ccc} M_{\mathrm{cor}} & = & M_{\mathrm{corF}}M_{\mathrm{dcor}}M_{\mathrm{corB}}\\ & = & M_{\mathrm{sphere}}\left(r_{\mathrm{corF}},n_{\mathrm{cor}},n_{\mathrm{air}}\right)M_{\mathrm{unif}}\left(d_{\mathrm{cor}}\right)M_{\mathrm{sphere}}\left(r_{\mathrm{corB}},n_{\mathrm{aq}},n_{\mathrm{cor}}\right)\\ & = & \left[\begin{array}{cc} \frac{d_{\mathrm{cor}}\left(-n_{\mathrm{cor}}+n_{\mathrm{aq}}\right)}{r_{\mathrm{corB}}n_{\mathrm{cor}}}+1 & \frac{d_{\mathrm{cor}}n_{\mathrm{aq}}}{n_{\mathrm{cor}}}\\ \frac{-n_{\mathrm{air}}+n_{\mathrm{cor}}}{r_{\mathrm{corF}}n_{\mathrm{air}}}+\frac{\left(-n_{\mathrm{cor}}+n_{\mathrm{aq}}\right)\left(\frac{d_{\mathrm{cor}}\left(-n_{\mathrm{air}}+n_{\mathrm{cor}}\right)}{r_{\mathrm{corF}n_{\mathrm{air}}}}+\frac{n_{\mathrm{cor}}}{n_{\mathrm{air}}}\right)}{r_{\mathrm{corB}}n_{\mathrm{cor}}} & \frac{n_{\mathrm{aq}}\left(\frac{d_{\mathrm{cor}}\left(-n_{\mathrm{air}}+n_{\mathrm{cor}}\right)}{r_{\mathrm{corF}}n_{\mathrm{air}}}+\frac{n_{\mathrm{cor}}}{n_{\mathrm{air}}}\right)}{n_{\mathrm{cor}}} \end{array}\right] \end{array}$$

(A.4) $$\begin{array}{ccc} M_{\mathrm{ACD}} & = & M_{\mathrm{unif}}\left(d_{\mathrm{ACD}}\right)=\left[\begin{array}{cc} 1 & d_{\mathrm{ACD}}\\ 0 & 1 \end{array}\right]\\ M_{\mathrm{lens}} & = & M_{\mathrm{lensF}}M_{\mathrm{dlens}}M_{\mathrm{lensB}}\\ & = & M_{\mathrm{sphere}}\left(r_{\mathrm{lensF}},n_{\mathrm{lens}},n_{\mathrm{aq}}\right)M_{\mathrm{unif}}\left(d_{\mathrm{lens}}\right)M_{\mathrm{sphere}}\left(r_{\mathrm{lensB}},n_{\mathrm{vitr}},n_{\mathrm{lens}}\right)\\ & = & \left[\begin{array}{cc} \frac{d_{\mathrm{lens}}\left(-n_{\mathrm{lens}}+n_{\mathrm{vitr}}\right)}{r_{\mathrm{lensB}}n_{\mathrm{lens}}}+1 & \frac{d_{\mathrm{lens}}n_{\mathrm{vitr}}}{n_{\mathrm{lens}}}\\ \frac{-n_{\mathrm{aq}}+n_{\mathrm{lens}}}{r_{\mathrm{lensF}}n_{\mathrm{aq}}}+\frac{\left(-n_{\mathrm{lens}}+n_{\mathrm{vitr}}\right)\left(\frac{d_{\mathrm{lens}}\left(-n_{\mathrm{aq}}+n_{\mathrm{lens}}\right)}{r_{\mathrm{lensF}}n_{\mathrm{aq}}}+\frac{n_{\mathrm{lens}}}{n_{\mathrm{aq}}}\right)}{r_{\mathrm{lensB}}n_{\mathrm{lens}}} & \frac{n_{\mathrm{vitr}}\left(\frac{d_{\mathrm{lens}}\left(-n_{\mathrm{aq}}+n_{\mathrm{lens}}\right)}{r_{\mathrm{lens}F}n_{\mathrm{aq}}}+\frac{n_{\mathrm{lens}}}{n_{\mathrm{aq}}}\right)}{n_{\mathrm{lens}}} \end{array}\right] \end{array}$$

(A.5) $$\begin{array}{c} M_{\mathrm{vitr}}=M_{\mathrm{unif}}\left(d_{\mathrm{vitr}}\right)=\left[\begin{array}{cc} 1 & d_{\mathrm{vitr}}\\ 0 & 1 \end{array}\right]\; \end{array}$$

(A.6) $$\begin{array}{c} M_{\mathrm{eye}}=M_{\mathrm{cor}}M_{\mathrm{ACD}}M_{\mathrm{lens}}M_{\mathrm{vitr}},\; \end{array}$$

with the values for the Escudero-Sanz–Navarro^21^ eye model at a wavelength of 543 nm as shown in the Table.

**Table:**

Ocular structures for the Escudero-Sanz–Navarro eye model		
at a wavelength of 543 nm		
*r*_corF_	Anterior corneal curvature	−7.74 mm
*r*_corB_	Posterior corneal curvature	−6.50 mm
*r*_lensF_	Anterior lens curvature	−10.20 mm
*r*_lensB_	Posterior lens curvature	+6.00 mm
*d*_cor_	Corneal thickness	0.55 mm
*d*_ACD_	Anterior chamber depth	3.05 mm
*d*_lens_	Lens thickness	4.00 mm
*d*_vitr_	Vitreous depth	16.32 mm
*n*_air_	Refractive index of air	1
*n*_cor_	Refractive index of the cornea	1.3777
*n*_aq_	Refractive index of the aqueous	1.3391
*n*_lens_	Refractive index of the lens	1.4222
*n*_vitr_	Refractive index of the vitreous	1.3377

For the pseudophakic eye, the same model can be used but with the thickness, radii, and refractive index of the IOL instead of the crystalline lens. For an eye with a pIOL, an additional **M**_pIOL_ is introduced, which describes both the pIOL and *d*_aq2_, the aqueous between the pIOL and crystalline lens:

(A.7) $$\begin{array}{ccc} \;\;\;\;\;M_{\mathrm{pIOL}} & = & M_{\mathrm{pIOLF}}M_{\mathrm{dpIOL}}M_{\mathrm{pIOLB}}M_{\mathrm{daq}2}\\ & = & M_{\mathrm{sphere}}\left(r_{\mathrm{pIOLF}},n_{\mathrm{pIOL}},n_{\mathrm{aq}}\right)M_{\mathrm{unif}}\left(d_{\mathrm{pIOL}}\right)\\ & & M_{\mathrm{sphere}}\left(r_{\mathrm{pIOLB}},n_{\mathrm{aq}},n_{\mathrm{pIOL}}\right)M_{\mathrm{unif}}\left(d_{\mathrm{daq}2}\right) \end{array}$$

(A.8) $$M_{\mathrm{eyepioL}}=M_{\mathrm{cor}}M_{\mathrm{ACD}}M_{\mathrm{pIOL}}M_{\mathrm{lens}}M_{\mathrm{vitr}}.$$

#### Camera Model

The camera is modeled by two thin lenses, a fixed condenser lens (**M**_cond_) and a variable focusing lens (**M**_foc_), with *r*_foc_ its radius of curvature. The imaging plane is positioned at a distance *d*_CCD_ from these lenses. The imaging plane is positioned at the focal length (*F*_cond_) of the condenser lens so it is in focus for an emmetropic eye. The refractive indexes of both lenses are chosen to be 1.5.

(A.9) $$\begin{array}{c} \begin{array}{ccc} M_{\mathrm{cond}} & = & \left[\begin{array}{cc} 1 & 0\\ -1/F_{\mathrm{cond}} & 1 \end{array}\right]\\ & = & \left[\begin{array}{cc} 1 & 0\\ \left(1.5-1\right)/-F_{\mathrm{cond}} & 1.5 \end{array}\right]\\ & & \left[\begin{array}{cc} 1 & 0\\ \left(1/1.5-1\right)/F_{\mathrm{cond}} & 1/1.5 \end{array}\right]\\ & = & M_{\mathrm{sphere}}\left(-F_{\mathrm{cond}},1.5,1\right)M_{\mathrm{sphere}}\left(F_{\mathrm{cond}},1,1.5\right) \end{array}\;\;\;\; \end{array}$$

(A.10) $$M_{\mathrm{foc}}=M_{\mathrm{sphere}}\left(-r_{\mathrm{foc}},1.5,1\right)M_{\mathrm{sphere}}\left(r_{\mathrm{foc}},1,1.5\right)$$

(A.11) $$M_{\mathrm{dCCD}}=M_{\mathrm{unif}}\left(d_{\mathrm{CCD}}\right)=M_{\mathrm{unif}}\left(F_{\mathrm{cond}}\right)$$

As this is a highly simplified model of a fundus camera, especially in terms of the optical effects when adjusting its focus, a first-order correction term (**M**_a1_) is added, which is proportional to the focusing power in diopters:

(A.12) $$M_{a1}=\left[\begin{array}{cc} 1+a1/r_{\mathrm{foc}} & 0\\ 0 & \frac{1}{1+a1/r_{\mathrm{foc}}} \end{array}\right]$$

making the total camera model:

(A.13) $$\begin{array}{ccc} M_{\mathrm{camera}} & = & M_{a1}M_{\mathrm{dCCD}}M_{\mathrm{foc}}M_{\mathrm{cond}}\\ & = & \left[\begin{array}{cc} -\frac{F_{\mathrm{cond}}\left(r_{\mathrm{foc}}+a1\right)}{r_{\mathrm{foc}}^{2}} & \frac{F_{\mathrm{cond}}\left(r_{\mathrm{foc}}+a1\right)}{r_{\mathrm{foc}}}\\ -\frac{F_{\mathrm{cond}}+r_{\mathrm{foc}}}{F_{\mathrm{cond}}\left(r_{\mathrm{foc}}+a1\right)} & \frac{r_{\mathrm{foc}}}{r_{\mathrm{foc}}+a1} \end{array}\right]. \end{array}$$

### Complete Model

The complete eye–camera setup can be described by

(A.14) $$M_{\mathrm{system}}=M_{\mathrm{camera}}M_{\mathrm{unif}}\left(d_{\mathrm{eyecam}}\right)M_{\mathrm{eye}},$$

where *d*_eyecam_ is the distance between the anterior corneal surface and the fundus camera. When the ocular geometry and the two camera-specific constants *F*_cond_ and *a*1 are known, the only unknown variable of **M**_system_ is the power of the focusing lens, expressed as the radius of curvature *r*_foc_.

### Some Useful Relations

For any paraxial system described by the matrix

(A.15) $$M_{ABCD}=\left[\begin{array}{cc} A & B\\ C & D \end{array}\right],$$

the system is in focus when *B* = 0. In this case, *A* is equivalent to the magnification of the system. Moreover, when *D* = 0, all light rays exit the system parallel to the optical axis, and no image is formed. Furthermore, the input and output nodal point locations, *N*_in_ and *N*_out_, can be calculated by *N*_in_ = (*D* – 1)/*C* and *N*_out_ = $(\frac{n_{i}}{n_{o}}-A)/C$, where *n_i_* and *n_o_* are the respective refractive indices of the input and output media. *N*_in_ and *N*_out_ are relative to the input and output planes, respectively, and distances measured after these planes are considered positive.

When the matrix **M**_eye_ of Equation A.6 is used to calculate the nodal points of the eye, one has to consider that in ophthalmology the location of these points is commonly expressed as a distance relative to the anterior cornea (instead of the input and output plane) and that **M**_eye_ describes an “inverse” eye where the light travels from the retina to the cornea. From this, it can be deduced that *N*_1_ = (*A* – *n*_vitr_)/*C* and that *N*_2_ = *AL* – (*D* – 1)/*C*, where *AL* is the axial length of the eye.
