# Supplementary material for: Correction Method for Optical Scaling of Fundoscopy Images: Development, Validation, and First Implementation
Source: Invest Ophthalmol Vis Sci. 2024 Jan 25;65(1):43. doi: 10.1167/iovs.65.1.43 (PMC10829800; doi:10.1167/iovs.65.1.43)
Supplement: Supplement 1 [file iovs-65-1-43_s001.pdf]

Supplementary materials

Calibration phantom eye setups:

Lens 1	Lens 2	Camera - pinhole distance (mm)	Pinhole - lens 1 distance (mm)	Lens 1 - lens 2 distance (mm)	Lens 2 - calibration object distance (mm)	Axial length (mm)	Refraction error (D)	Measured magnification
LB_1471	LB_1945	49.6	2.9	3.0	29.7	40.7	3.8	0.70
LB_1471	LB_1945	49.6	2.9	3.0	31.7	42.7	2.4	0.67
LB_1471	LB_1945	49.6	2.9	3.0	33.7	44.7	1.0	0.66
LB_1471	LB_1945	49.6	2.9	3.0	35.7	46.7	-0.2	0.64
LB_1471	LB_1945	49.6	2.9	3.0	37.7	48.7	-1.3	0.63
LB_1471	LB_1945	49.6	2.9	3.0	39.7	50.7	-2.4	0.61
LB_1471	LB_1945	49.6	2.9	3.0	41.7	52.7	-3.4	0.60
LB_1471	LB_1945	49.6	2.9	3.0	43.7	54.7	-4.3	0.57
LB_1471	LB_1945	49.6	2.9	3.0	45.4	56.4	-5.0	0.56

Test phantom eye setups:

Lens 1	Lens 2	Camera - pinhole distance (mm)	Pinhole - lens 1 distance (mm)	Lens 1 - lens 2 distance (mm)	Lens 2 - calibration object distance (mm)	Axial length (mm)	Refraction error (D)	Measured magnification	Model magnification
LE_1234	LB_1945	55.3	2.2	5.3	56.4	68.1	1.05	0.40	0.40
LE_1234	LB_1945	55.3	2.2	5.3	65.9	77.6	-0.96	0.36	0.36
LE_1234	LB_1056	50.3	2.2	5.2	65.2	76.6	0.03	0.36	0.36
LE_1234	LB_1056	50.3	2.2	5.2	60.1	71.5	1.03	0.37	0.38
LE_1234	LB_1056	52.3	2.2	5.2	70.7	82.1	-0.94	0.34	0.34
LE_1234	LB_1779	50.3	2.2	5.3	62.7	74.1	1.03	0.36	0.36
LE_1234	LB_1779	50.3	2.2	5.3	57.3	68.6	2.18	0.38	0.39
LE_1234	LB_1779	50.3	2.2	5.3	69.1	80.4	-0.11	0.34	0.34
LE_1234	LB_1779	50.3	2.2	5.3	74.6	85.9	-0.97	0.32	0.32
LE_1156	LB_1945	50.2	2.3	6.3	71.5	84.0	0.05	0.32	0.33
LE_1156	LB_1945	50.2	2.3	6.3	65.6	78.0	1.06	0.34	0.35
LE_1156	LB_1945	50.2	2.3	6.3	78.3	90.7	-0.95	0.31	0.31
LE_1156	LB_1056	50.2	2.3	6.4	77.2	89.5	0.06	0.31	0.31
LE_1156	LB_1056	50.2	2.3	6.4	70.4	82.7	1.07	0.32	0.33
LE_1156	LB_1056	50.2	2.3	6.4	85.2	97.5	-0.96	0.29	0.29
LE_1156	LB_1779	50.2	2.3	6.5	81.8	94.0	0.02	0.29	0.29
LE_1156	LB_1779	50.2	2.3	6.5	74.3	86.5	1.02	0.31	0.31
LE_1156	LB_1779	50.2	2.3	6.5	90.5	102.7	-0.96	0.27	0.27
LE_1104	LB_1945	50.4	2.1	7.7	80.7	94.3	0.02	0.29	0.30
LE_1104	LB_1945	50.4	2.1	7.7	73.5	87.1	1.01	0.31	0.32
LE_1104	LB_1945	50.4	2.1	7.7	89.4	103.0	-0.99	0.27	0.28
LE_1104	LB_1056	50.4	2.1	7.8	88.2	101.7	0.00	0.27	0.27

LE_1104	LB_1056	50.4	2.1	7.8	79.6	93.1	1.00	0.29	0.29
LE_1104	LB_1779	50.4	2.1	7.8	93.8	107.2	0.01	0.25	0.26
LE_1104	LB_1779	50.4	2.1	7.8	84.1	97.5	1.02	0.27	0.28
LE_1234	LB_1056	50.3	2.2	5.2	65.2	76.6	0.03	0.35	0.36
LE_1234	LB_1056	50.3	2.2	5.2	60.1	71.5	1.03	0.37	0.38
LE_1234	LB_1056	50.3	2.2	5.2	70.7	82.1	-0.94	0.34	0.34
LE_1234	LB_1056	50.3	2.2	5.2	85.3	96.7	-2.97	0.29	0.30
LE_1234	LB_1056	50.3	2.2	5.2	63.8	75.2	0.29	0.36	0.36
LE_1234	LB_1056	50.3	2.2	5.2	55.1	66.5	2.17	0.39	0.40
LE_1234	LB_1056	50.3	2.2	5.2	47.8	59.2	4.14	0.43	0.44
LE_1156	LB_1945	50.2	2.3	6.3	71.5	84.0	0.05	0.33	0.33
LE_1156	LB_1945	50.2	2.3	6.3	78.3	90.7	-0.95	0.31	0.31
LE_1156	LB_1945	50.2	2.3	6.3	65.6	78.0	1.06	0.35	0.35
LE_1156	LB_1945	50.2	2.3	6.3	122.2	134.6	-5.08	0.22	0.22
LE_1156	LB_1945	50.2	2.3	6.3	95.5	107.9	-2.95	0.27	0.27
LE_1156	LB_1945	50.2	2.3	6.3	59.6	72.0	2.24	0.37	0.37
LE_1156	LB_1945	50.2	2.3	6.3	51.2	63.6	4.24	0.40	0.41
LE_1234	LB_1945	50.3	2.2	5.1	140.9	152.4	-8.27	0.20	0.20
LE_1234	LB_1056	50.3	2.2	5.2	141.1	152.5	-7.27	0.20	0.20

Lens properties:

Name	Type	Centre thickness	Edge thickness	Diameter	Power (D)	Anterior curvature (mm)	Posterior curvature (mm)	Refractive index
LB_1409	biconvex	2.2	2	25.4	1	1029.8	-1029.8	1.515
LB_1869	biconvex	2.3	2	25.4	2	514.7	-514.7	1.515
LE_1234	convex-concave	3.6	2	25.4	10	82.2	32.1	1.515
LE_1156	convex-concave	3.3	2	25.4	8	106.9	40.6	1.515
LE_1104	convex-concave	3.1	2	25.4	6.7	131.6	49.1	1.515
LE_1202	convex-concave	2.8	2	25.4	5	182.2	66.2	1.515
LB_1945	biconvex	2.8	2	25.4	5	205.6	-205.6	1.515
LB_1056	biconvex	2.6	2	25.4	4	257.1	-257.1	1.515
LB_1779	biconvex	2.5	2	25.4	3.3	308.6	-308.6	1.515
LB_1471	biconvex	5.2	2	25.4	20	50.6	-50.6	1.515
